# Capturing the inflammatory landscape within kidney compartments of human diabetic kidney disease: a digital spatial profiling study

**DOI:** 10.3389/fendo.2026.1744430

**Published:** 2026-02-02

**Authors:** Khaled M. Elhusseiny, Farha G. Deceus, Lynn D. Cornell, Xiaohui Bian, Yaohua Ma, Jennifer M. Kachergus, E. Aubrey Thompson, LaTonya J. Hickson

**Affiliations:** 1 Division of Nephrology and Hypertension, Department of Medicine, Mayo Clinic, Jacksonville, FL, United States; 2 Department of Internal Medicine, Mayo Clinic, Scottsdale, AZ, United States; 3 Department of Laboratory Medicine and Pathology, Mayo Clinic, Rochester, MN, United States; 4 Department of Pathology and Laboratory Medicine, University of Calgary, Calgary, AB, Canada; 5 Department of Nephrology, Shengjing Hospital of China Medical University, Shenyang, China; 6 Division of Biomedical Statistics, Mayo Clinic, Jacksonville, FL, United States; 7 Department of Cancer Biology, Mayo Clinic, Jacksonville, FL, United States

**Keywords:** inflammation, macrophages, pathology, T cells, tubulointerstitial nephritis

## Abstract

**Objective:**

To quantitatively examine immune cell markers and spatial distribution in human diabetic kidney disease (DKD) to enhance understanding of the inflammatory landscape contributing to injury. Maladaptive inflammation is an underrecognized contributor to DKD pathogenesis and progression and remains undertreated.

**Patients and methods:**

NanoString GeoMx™ Digital Spatial Profiling technology targeted antibodies labeled with unique oligonucleotide barcode in kidney biopsy [DKD (n=5), tubulointerstitial nephritis (TIN; n=4), and normal (n=2)] with regions of interest selection of compartments (glomeruli, tubules, interstitium). Inflammation-related proteins were analyzed with differential expression through linear mixed modeling.

**Results:**

Compared to normal tissue, inflammatory cell surface protein markers were increased in DKD tubules and interstitium. Markers of T cells (CD4, CD44), macrophages (CD68; proinflammatory), and antigen-presenting cells (APCs; CD40 and CD11c) were increased across all DKD compartments (vs. normal). Macrophage (CD163; prorepair) marker was increased in DKD tubules and interstitium (vs. normal). Fewer differences were observed in glomeruli for normal vs. DKD or TIN vs. DKD groups. CD66b+ (granulocytes) cell marker was higher in DKD (vs. TIN). As expected, TIN had higher levels of T cell and macrophage markers in tubules and interstitium (vs. DKD). Interestingly, CD34, a hematopoietic stem cell and endothelial cell marker, was lower in DKD tubules and interstitium (vs. normal) but higher in DKD (vs. TIN).

**Conclusion:**

NanoString GeoMx DSP technology may fulfil a role in enhancing the understanding the inflammatory landscape engaged in DKD pathogenesis as well as measuring response to therapy. Moreover, additional investigations of CD34 progenitor cell depletion in DKD may be warranted.

## Highlights

• What was known: Maladaptive inflammation is an underrecognized contributor to diabetic kidney disease (DKD) injury and progression and remains undertreated. Limited information is available which quantifies immune cell infiltration in diabetic kidney compartments and it is unclear how the inflammatory landscape compares to tubulointerstitial nephritis (TIN) in humans.
• This study adds: Comparisons of immune markers and spatial distribution in diabetic kidney disease, normal, and tubulointerstitial nephritis tissue are rare. NanoString GeoMx Digital Spatial Profiling technology helped identify that inflammatory cell infiltration is higher across all compartments in DKD compared to normal tissue and more closely mirrors the inflammatory landscape of TIN.
• Potential impact: NanoString GeoMx DSP technology may aid in furthering understanding of DKD pathogenesis as well as measuring response to therapy. Renewed focus on therapeutic targeting of pro-inflammatory pathways may improve DKD outcomes. Additionally, investigations focusing on CD34, a hematopoietic stem cell and endothelial cell marker, progenitor depletion in DKD may be warranted.

## Introduction

Diabetes mellitus (DM) is the leading cause of kidney failure, and with the global prevalence of DM predicted to rise to 12.2% in 2045, corresponding increases in diabetic kidney disease (DKD) are anticipated (1, 2). Several pathogenic processes contribute to DKD, including hyperglycemia, advanced glycation end-products (AGE), oxidative stress, renin-angiotensin-aldosterone system (RAAS) dysregulation, cellular senescence, and abnormal intracellular metabolism, which lead to chronic sterile inflammation, vascular and tissue damage, and resultant fibrosis (3). Several therapies currently target DKD processes, such as strict glycemic control, renin-angiotensin-aldosterone system inhibitors, sodium-glucose cotransporter inhibitors, glucagon-like peptide-1 agonists, dipeptidyl peptidase-4 inhibitors, and endothelin A antagonists (4, 5). However, immune cell infiltration is commonly found in clinical DKD biopsies, yet conventional therapy fails to focus treatment on inflammation as a key component of DKD pathogenesis (6–9). Hence, gaining a better understanding of the inflammatory landscape in DKD may aid in development of effective therapies.

Both the adaptive and innate immune systems contribute to the inflammatory response in DKD (10, 11). Early in DKD, increased expression of proinflammatory molecules, including cytokines, chemokines (12), monocyte chemoattractant protein-1 (MCP-1) (13), intercellular adhesion molecule-1 (ICAM-1) (14), and macrophage inflammatory protein-1 (MIP-1) (15) culminate in macrophage infiltration of the glomeruli and tubulointerstitium. Previous analysis showed heterogenous macrophage infiltration with increased M-1 proinflammatory phenotype over time (16). Importantly, macrophage infiltration and abundance are associated with DKD progression (6, 17). Diabetic mice deficient in ICAM-1 (ICAM-1^-^/^-^), a macrophage chemoattractant and adhesion molecule, had lower kidney macrophage infiltration and albuminuria compared to ICAM-1^+^/^+^ controls (14). Macrophage chemokine receptor (CCR1, CCR2) inhibition was associated with reduced glomerulosclerosis in diabetic mice (18). As part of adaptive immunity, T cells are also implicated in DKD injury, with infiltration associated with higher proteinuria (19, 20).

While immune cells infiltrate all kidney compartments, including the glomeruli, tubules, and interstitium, it is not a homogenous process (21). Both macrophages (10, 21, 22) and T cells (20, 23) are dispersed across compartments in DKD, yet macrophages make up the most abundant cell type (17, 24). In a type 2 diabetes model, macrophage accumulation and activation associated with increased macrophage chemokine markers, kidney injury and fibrosis in 8 month old mice (17), and their presence particularly in the interstitium has been correlated with albuminuria, serum creatinine, and interstitial fibrosis (21, 25). Similarly, T cell infiltration, primarily in the interstitium, has been identified in humans and mouse models of DKD (19, 21). DKD pathogenesis, including interstitial fibrosis and resulting progressive kidney dysfunction, may be primarily driven by tubulointerstitial inflammation (12, 26).

Newer techniques for identifying immune cells, such as single-cell RNA sequencing (scRNA-seq), have reinforced the presence of proinflammatory response and leukocyte infiltration in DKD (16, 27). However, spatial multiomics, including spatial proteomics and spatial transcriptomics, has received increasing attention since its discovery in 2016, particularly in cancer biology (28). Its use in understanding the tumor microenvironment and associated disease progression and treatment response holds great promise (29, 30); yet, there has been minimal application in DKD. While the patterns of immune cell infiltration in human DKD are traditionally examined through immune staining or recently in scRNA-seq studies, few studies have quantitatively examined the immune markers and spatial distribution with comparisons to normal kidney tissue and densely inflamed kidney tissue, such as tubulointerstitial nephritis (TIN) (16, 27).

We tested the hypothesis that human kidney tissue in DKD possesses an inflammatory profile distinct from normal histology but less dissimilar to TIN, therein warranting development of novel strategies to target inflammation. In these studies, we examine the kidney immune landscape in kidney compartments using novel NanoString GeoMx™ Digital Spatial Profiling (DSP) technology; a spatially resolved, high-plex protein and RNA profiling assay which allows for quantification of desired biomarkers within user-defined regions of interest (ROIs) (31). DSP is advantageous over traditional molecular profiling modalities given its ability to yield robust data on the morphologic and quantitative heterogeneity of immune profiles using minimal, archival tissue samples (31). To date, few published studies applied NanoString GeoMx DSP to assess the protein profiles in human medical kidney disease, such as ANCA-associated glomerulonephritis (32) and focal necrotizing glomerulonephritis (33), but none evaluated the inflammatory signature in DKD. To fill this gap, we assessed the differential abundance of inflammatory cell surface proteins in kidney biopsy tissue from patients with normal histology, DKD, and TIN.

## Materials and methods

This study was conducted with Mayo Clinic Institutional Review Board (IRB) approval. Kidney biopsy tissue specimens were identified for patients with normal kidney histology, DKD, and TIN at Mayo Clinic in Rochester, Minnesota. Limited deidentified data were obtained, including age, gender, diabetes mellitus diagnosis, and kidney function parameters, including serum creatinine and estimated glomerular filtration rate (eGFR CKD-EPI). Normal histology specimens were native biopsy specimens from adults with thin basement membrane nephropathy (n=1) or normal histology without glomerular basement membrane thinning (n=1). Patients with DKD were diagnosed based on clinicopathologic criteria, including documented history of diabetes mellitus with persistent albuminuria or reduced eGFR. Biopsies showing DKD were selected that demonstrated nodular diabetic glomerulosclerosis and moderate interstitial fibrosis and tubular atrophy (IFTA). TIN cases were diagnosed in non-diabetic patients based on biopsy-proven interstitial inflammation with tubular injury, in the absence of primary glomerular disease, and supported by clinical context consistent with known etiologies of TIN, including proton pump inhibitor–associated chronic allergic TIN, IgG4-related TIN, granulomatous TIN (ANCA-associated), immune checkpoint inhibitor-associated TIN, and HIV-associated chronic TIN. Biopsies within the DKD and TIN groups represented a spectrum of disease severity reflective of real-world clinical presentation.

### GeoMx digital spatial profiling

Our use of protein-based DSP has been previously described (34). Briefly, FFPE tissue slides were deparaffinized from core needle kidney biopsy samples. Using NanoString GeoMx DSP technology, slides were stained with fluorescently labeled antibodies. Antibodies were labeled with a unique oligonucleotide barcode attached by a UV-labile cross-linker, directed against 77 targets. Immunofluorescent antibodies against pan-cytokeratin (epithelium), CD45 (leukocytes), and CD68 (macrophage) plus the nuclear dye (STYO13) were used. Twelve regions of interest (ROIs) were obtained from each biopsy slide to capture glomeruli and tubulointerstitial regions in a randomized fashion under nephrologist and pathologist guidance. Of each ROI quantification, the tubular segment was identified as cytokeratin-positive, while the interstitial segment was identified as cytokeratin-negative/STYO13-positive (**Supplementary Figure 1**). Oligonucleotides bound to biomarkers of interest were quantified using the nCounter technology upon liberation by UV laser irradiation of computer-defined masks. Out of the 77 target proteins, 29 were selected for further analysis to focus on the innate and adaptive immunity in the kidney.

### Histological staining

Immunohistochemical staining for immune cell markers was performed. Antibodies for markers of T cells, including identification (CD3) and activation (CD45RO) and macrophage marker CD68 were selected.

### Statistical analysis

Data were normalized to the geometric mean of three housekeeping proteins (GAPDH, histone H3, S6). A linear mixed model was used to assess the differential expression of proteins using Log_2_ fold change (Log_2_FC). A two-sided p-value was considered significant if <0.05. Forest and volcano plot graphs were created using GraphPad Prism Version 10. For the forest plots, p values of <0.001 were converted to 0.001 and plotted as -Log10 value of 3.

## Results

### Baseline characteristics

Patient baseline characteristics are summarized in **Table 1**. Kidney biopsy specimens were obtained from individuals with normal kidney function/histology (n=2), DKD (n=5), and TIN (n=4). Fifty to 60% of all groups were female. Mean age was lowest in the normal group (39 ± 2 years) followed by DKD (52 ± 10) and TIN (68.3 ± 12.8). As anticipated, kidney function was highest in normal histology (eGFR 115 ± 1 mL/min/1.73m^2^), then followed by DKD (44 ± 22) and TIN (26 ± 21) groups. Only DKD patients had diabetes mellitus.

**Table 1:** Baseline characteristics.

Characteristic	Normal (n=2)	DKD (n=5)	TIN (n=4)
Age, years	38.5 ± 2.1	52.2 ± 10.1	68.3 ± 12.8
Female, n (%)	1 (50)	3 (60)	2 (50)
Creatinine, mg/dL	0.75 ± 0.1	2.1 ± 1.6	3.9 ± 3.5
eGFR CKD-EPI, mL/min/1.73m^2^	114.5 ± 0.7	43.6 ± 21.8	25.8 ± 21
Diabetes mellitus, n (%)	0	(5) 100	0

Sample mean and standard deviation are given for continuous data.

Number (percentage) is given for categorical data.

DKD, diabetic kidney disease; TIN, Tubulointerstitial nephritis.

### Spatial analysis and tissue histology

**Figure 1:**
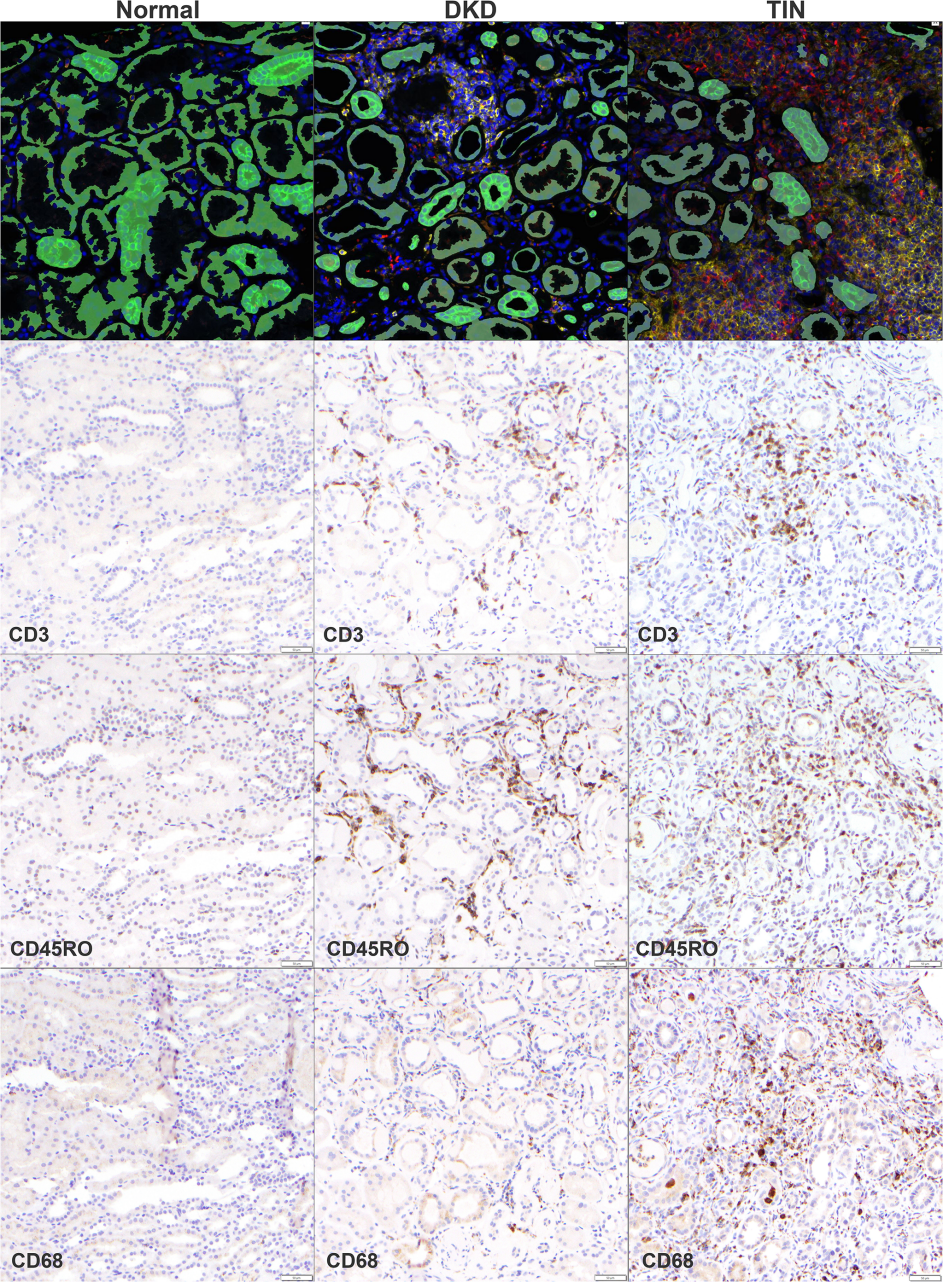
Digital spatial profiling and immunohistochemistry staining in normal, DKD, and TIN kidney tissue. Representative images are shown. For digital spatial profiling studies (Top row), fluorescent-labeled antibodies were directed against pan-cytokeratin (green; tubular epithelial cells), CD45 (yellow; leukocytes), CD68 (red; macrophages), and the blue nuclear dye (STYO13). For immunohistochemistry studies, antibodies were directed against CD3 (T cells), CD45RO (T cells), and CD68 (macrophage). DKD, diabetic kidney disease and TIN, tubulointerstitial nephritis.

Fluorescent-labeled antibodies were used to identify nephron structural units, including tubules (PanCK positive) and interstitium (SYTO13 positive, PanCK negative). While normal histology showed preserved structure, interstitial expansion and tubular dropout were prominent in DKD and TIN. Notably, a high abundance of staining for leukocytes (CD45, yellow) and macrophages (CD68, red) were observed in both DKD and TIN, **Figure 1**. To further characterize T cell and macrophage abundance, immunohistochemical stains were performed. As shown, CD3, CD45RO, and CD68 antibody staining were prevalent in TIN but also increased in DKD (vs. normal).

### Compartmental differences - DKD vs. normal

To better understand differences in the immune landscape between disease groups, comparisons were made across kidney compartments.

#### Glomerulus compartment

While many of the inflammatory protein markers trended higher in DKD glomeruli, most were not statistically different (vs. normal). However, three T cell markers were elevated in DKD, including CD4 (Log_2_FC 0.56, 95% CI 0.19, 0.92, p=0.006), CD44 (Log_2_FC 1.5, 95% CI 0.66, 2.33, p=0.02), and PD-1 (Log_2_FC 0.63, 95% CI 0.13, 1.13, p=0.02). In addition, antigen-presenting cell (APC) CD11c (Log_2_FC 0.7, 95% CI 0.24, 1.16, p=0.007) and macrophage CD68 (Log_2_FC 0.52, 95% CI 0.14, 0.89, p=0.01) markers were increased in DKD glomeruli, **Supplementary Figure 2**.

#### Tubular compartment

**Figure 2:**
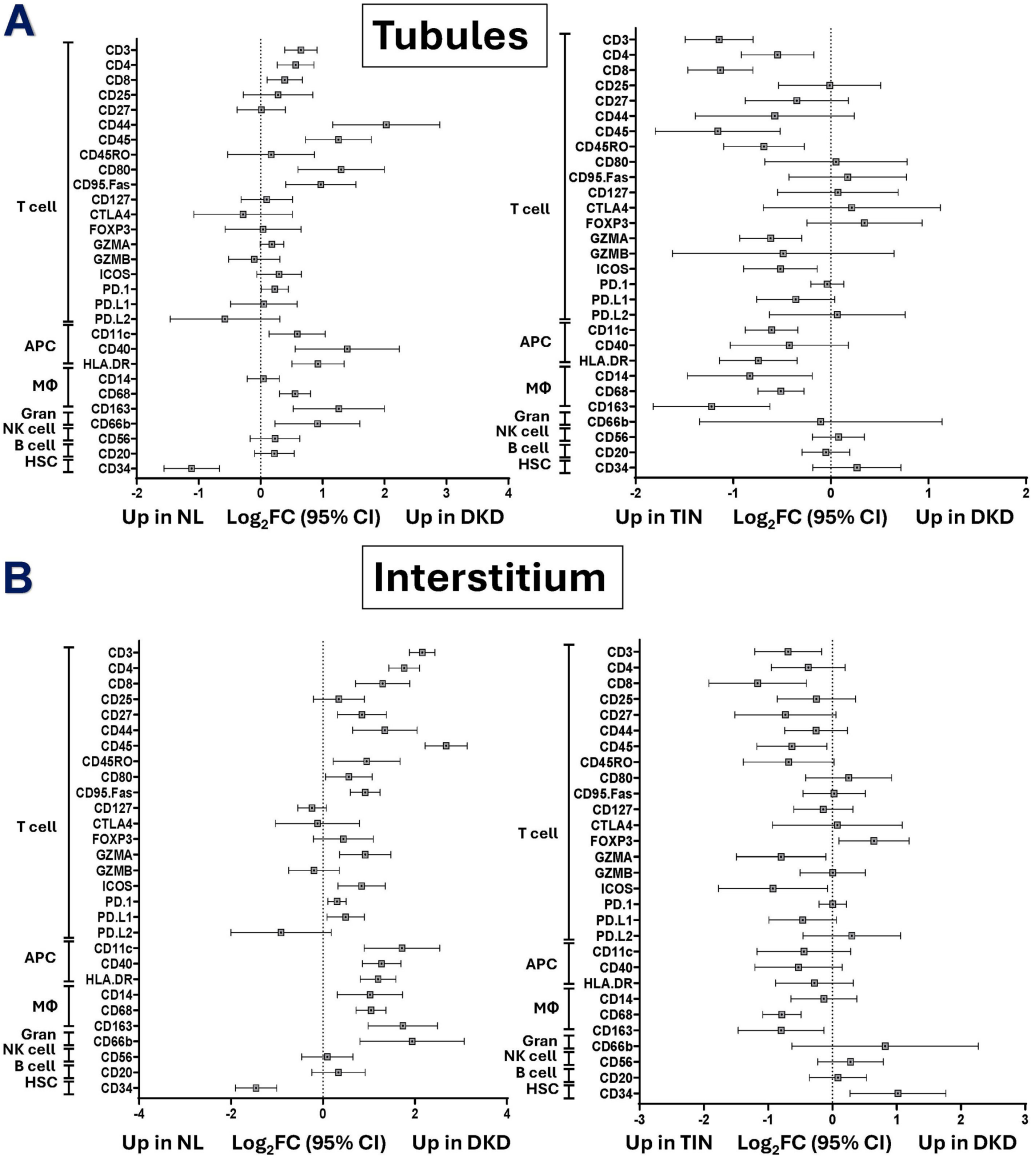
Immune cell marker distributions in the tubular and interstitial compartments of Normal, DKD, and TIN kidney tissue. Forest plots display differential expression of cell marker proteins. Data are displayed as Log2 Fold Change (Log2FC) and 95% confidence intervals (CIs). Comparison analyses are shown for the tubule compartments (DKD vs. NL [left] and DKD vs. TIN [right]) in Panel **(A)** Comparison analyses are shown for the interstitium compartments (DKD vs. NL [left] and DKD vs. TIN [right]) in Panel **(B)** APC, antigen-presenting cell; DKD, diabetic kidney disease; Gran, granulocyte; HSC, hematopoietic stem cell; MΦ, macrophage; NK cell, natural killer cell; NL, normal kidney tissue; and TIN, tubulointerstitial nephritis.

In contrast to the glomerular compartment, multiple T cell protein markers were significantly higher in DKD tubules vs. normal, including CD3, CD4, CD8, CD44, CD45, CD80, and CD95, **Figure 2A**. T cell PD-1 and GZMA markers trended higher (p=0.1). Similarly, macrophage (CD68 and CD163), APC (CD11c, CD40, HLA-DR), and granulocyte CD66b protein markers were more abundant in DKD tubules. However, the macrophage CD14 marker was not different.

#### Interstitial compartment

Differences in the inflammatory landscape were pronounced when DKD interstitium when compared to normal, **Figure 2B**. The T cell markers CD45 (Log_2_FC 2.68, 95% CI 2.22, 3.14, p<0.001), CD3, and CD4 had the highest fold change. Additionally, all APCs and macrophage cell markers were higher in DKD interstitium vs. normal.

#### Overlap across compartments for DKD vs. normal

**Figure 3:**
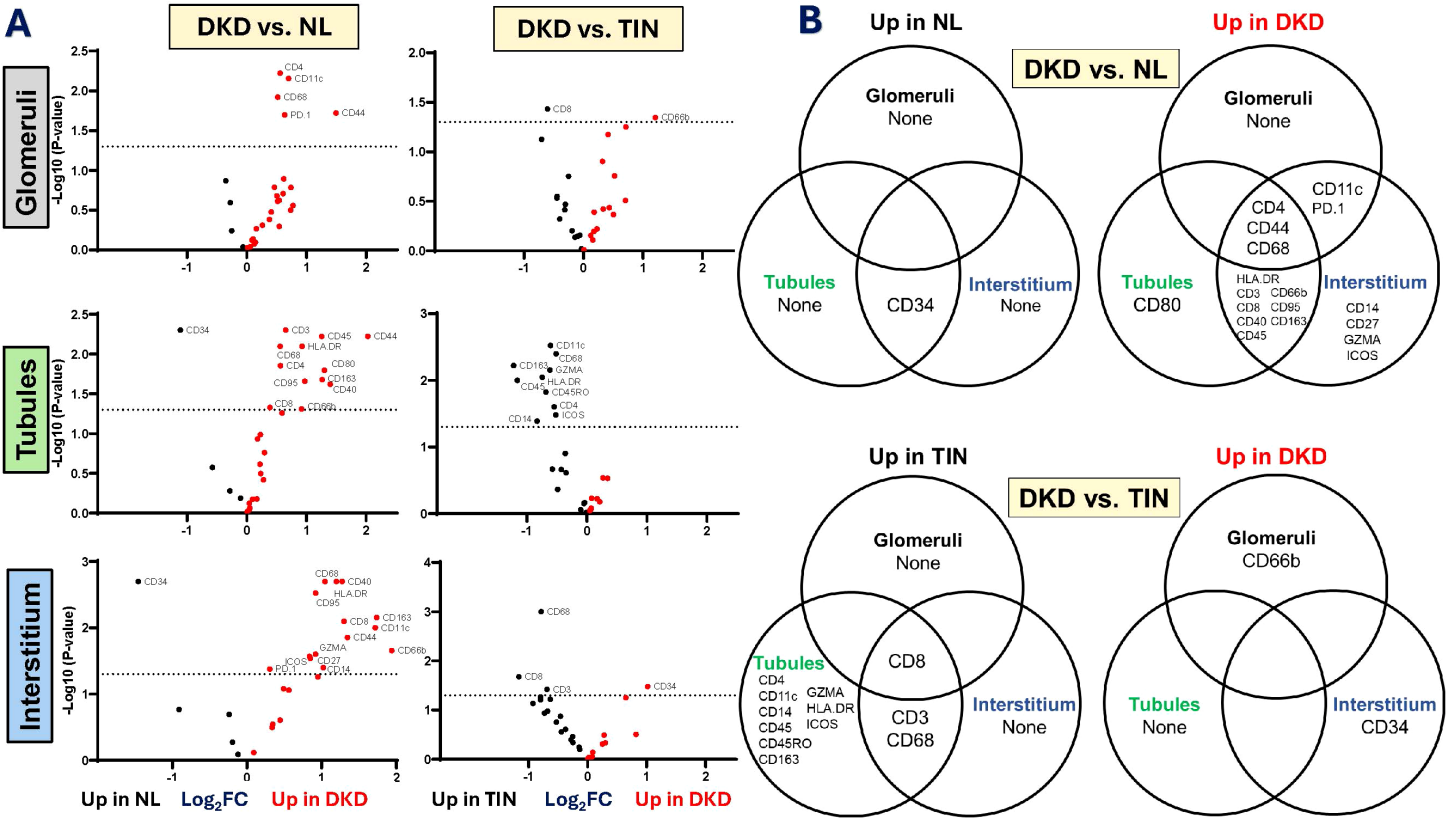
Immune cell marker differential distributions in the glomerular, tubular, and interstitial compartments of Normal, DKD, and TIN kidney tissue. Volcano plots display differential expression of cell marker proteins (Panel A). Venn diagrams show common and distinctive cell markers expression among the three compartments (Panel B). Data are displayed as Log2 Fold Change (Log2FC). Comparison analyses are shown for DKD vs. NL [left] and DKD vs. TIN [right]. DKD, diabetic kidney disease; NL, normal kidney tissue; and TIN, tubulointerstitial nephritis.

As shown in **Figure 3**, trends were identified for inflammatory protein distribution. Interestingly, HSC and endothelial cell marker CD34 was much lower in DKD interstitium and tubules compared to normal. Additionally, CD4, CD44, and CD68 were higher across all 3 compartments in DKD (vs. normal). Tubules and interstitium of DKD had higher abundance of inflammatory distribution. Notably, CD56, which is a marker for NK cells as well as activated T cells, monocytes, and dendritic cells (35), did not differ between DKD and normal for glomeruli, tubules, and interstitium compartments.

### Compartmental Differences – DKD vs. TIN

#### Glomerulus compartment

When glomeruli of DKD were compared to TIN, differences between inflammatory cell protein markers were minimal, **Supplementary Figure 2**. Granulocyte CD66b surface marker was higher (Log_2_FC 1.21, 95% CI 0.258, 2.15; p=0.045) in DKD glomeruli (vs. TIN). T regulatory protein marker FOXp3 trended higher in DKD (Log_2_FC 0.711, 95% CI 0.008, 1.415; p=0.06). However, TIN glomeruli had higher CD3+ and CD8+ T cell markers than DKD glomeruli (Log_2_FC -0.61, 95% CI -1.07, -0.15; p=0.04).

#### Tubular compartment

While multiple T cell protein markers were higher in TIN tubules (vs. DKD), including CD3, CD4, CD8, CD45, CD45RO, T-regulatory cell surface markers, including CD25 and FOXP3, were not different (Log_2_FC -0.012, 95% CI -0.54, 0.51; p=0.97) and (Log_2_FC 0.35, 95% CI -0.24, 0.93, p=0.3), respectively, **Figure 2**. However, most APC and macrophage markers were higher in the TIN tubules. Notably, no inflammatory markers were higher in DKD.

#### Interstitial compartment

Surprisingly, in contrast to the DKD tubule compartment, fewer inflammatory proteins were higher in the interstitial compartment of TIN (vs. DKD). CD3+ and CD8+ T cells were more abundant in TIN (Log_2_FC -0.69, 95% CI -1.21, -0.17; p=0.04) and (Log_2_FC -1.16, 95% CI -1.92, -0.4; p=0.02), respectively. Macrophage markers CD68+ and CD163+ were higher in both the tubules and interstitium of TIN (vs. DKD). Interestingly, only FOXP3 and CD34 were higher in DKD interstitium compared to TIN.

#### Overlap across compartments

As shown in **Figure 3**, T cell marker CD8 was higher in all three compartments. In the tubules, CD3, CD68, CD45, and CD163 were higher in the tubules of TIN. CD163 trended higher (p=0.05) but did not reach significance for TIN (vs. DKD). No immune markers were higher across all three compartments, but CD34 was higher in the interstitium and CD66b was higher in the glomeruli of DKD (vs. TIN).

### Immune cell type differences in normal, DKD, and TIN

**Figure 4:**
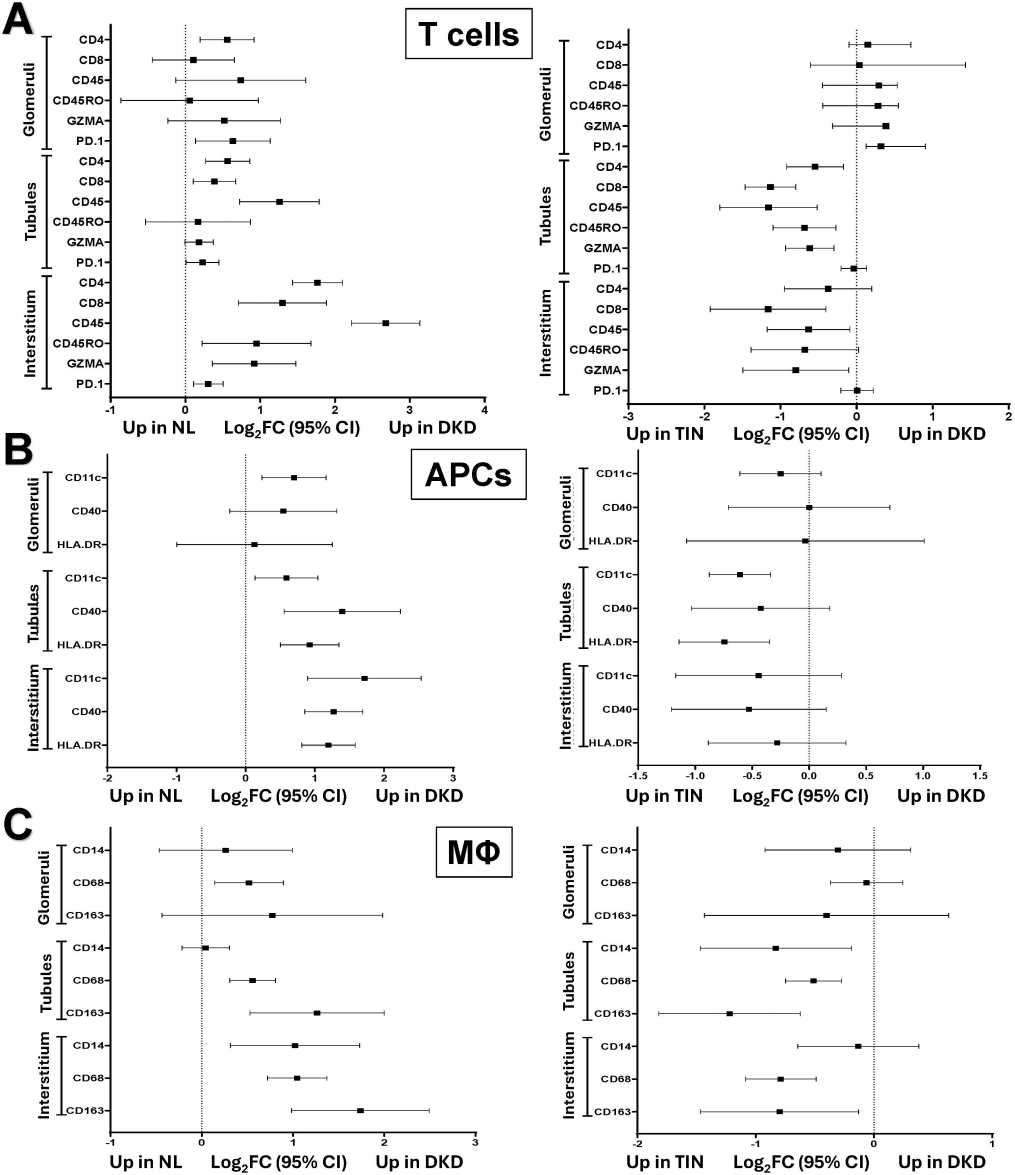
Immune cell type distribution by compartments in Normal, DKD, and TIN kidney tissue. Forest plots display differential expression of cell marker proteins. Data are displayed as Log2 Fold Change (Log2FC) and 95% confidence intervals (CIs). Comparison analyses are shown for T cells in glomerular, tubular, and interstitial compartments (DKD vs. NL [left] and DKD vs. TIN [right]) in Panel **(A)** Comparison analyses are shown for APC in glomerular, tubular, and interstitial compartments (DKD vs. NL [left] and DKD vs. TIN [right]) in Panel **(B)** Comparison analyses are shown for macrophage in glomerular, tubular, and interstitial compartments (DKD vs. NL [left] and DKD vs. TIN [right]) in Panel **(C)** APC, antigen-presenting cell; DKD, diabetic kidney disease; MΦ, macrophage; NL, normal kidney tissue; and TIN, tubulointerstitial nephritis.

To examine trends in immune cell marker prevalence, comparisons were made for T cells, APCs, and macrophages across compartments as well as between disease groups, **Figure 4**.

#### T cells

T cell surface marker proteins tended to differ significantly in the interstitium of DKD vs. normal. On the other hand, as expected, TIN tubules and interstitium had more T cell protein markers vs. DKD. CD4 marker was higher in DKD across all 3 compartments compared vs. normal. FOXP3 and CD25, T regulatory cell markers, were seldom different between normal, DKD and TIN, **Supplementary Figure 3**. However, FOXP3 trended higher in the DKD interstitium vs. TIN (Log_2_FC 0.65, 95% CI 1.102, 1.19, p=0.056).

#### Antigen-presenting cells

In glomeruli, most APC markers were not different in DKD vs. normal and DKD vs. TIN. However, APC markers were higher in both tubules and interstitium of DKD (vs. normal). Meanwhile, some levels were higher only in tubules of TIN (vs. DKD) with no differences for interstitium.

#### Macrophages

In DKD, CD68 was higher across all three compartments vs. normal. Yet, CD163 was increased in tubules and interstitium vs. normal. Interestingly, macrophage markers were not different between TIN and DKD glomeruli. However, the prevalence of macrophage markers CD68 and CD163 were (or trended) higher in TIN tubules and interstitium vs. DKD.

## Discussion

Our major study findings include the following: 1) DKD tissue has higher immune cell infiltration than normal tissue which is present across all three kidney compartments; 2) the primary sites of increased immune cell infiltration in DKD are the tubular and interstitial compartments which are enriched in macrophage and T cell markers; 3) the DKD inflammatory landscape closely mirrors TIN despite this being considered a primarily inflammatory condition. Previous immune profiling studies have compared DKD to other inflammatory nephropathies (36–38), usually glomerulonephritis, or to normal tissue (39). While DKD involves robust changes to the glomerulus eliciting nodular glomerulosclerosis, inflammation in DKD appears driven by the tubulointerstitium (6, 12, 18, 19, 40). To our knowledge, we are the first to compare immune cell infiltration in DKD kidney biopsy tissue to both normal tissue and tubulointerstitial nephritis. Moreover, we utilized a newer tool, NanoString GeoMx™ DSP technology for quantification. These studies provide further evidence that DKD is a proinflammatory condition with T cell and macrophage predominant cell types. Furthermore, HSC and endothelial cell marker CD34 abundance decreases with DKD and TIN.

Compared to normal kidney tissue, immune cell infiltration in DKD appears enriched in T cell CD4, antigen presenting cell (lymphocyte migration/activation) CD44, and macrophage CD68 markers which were also higher across all three compartments. When DKD was compared to TIN, an increased expression of granulocyte marker CD66b was observed. Previous studies reported no significant increase in glomerular leukocytes (26, 41). Yet, more recent work found increases in multiple mononuclear immune cell types in DKD glomeruli (25, 42). Furuta et al. (43) observed that macrophages would transiently increase early in DKD progression and fall in more advanced stages. The eGFR in our sampled DKD patients was in the moderate to severe CKD range so these individuals may have progressed past their glomerular leukocyte peak.

In DKD, most immune cell infiltration was found in the tubular and interstitial compartments. Compared to normal tissue, DKD showed elevated cell surface protein markers, including T cell (CD3, CD4, CD8, CD45, CD80, CD95, PD.1), macrophage (CD14, CD40, CD68, CD163), APC (CD11c, HLA.DR), and granulocyte (CD66b) markers. This finding was expected given numerous studies describing similar increases in tubulointerstitial leukocytes (12, 18, 19, 41, 44). As anticipated, TIN had higher leukocyte surface protein markers in the tubular and interstitial compartments compared to DKD; however, of the 29 immune cell surface markers analyzed, only 12 were significantly higher in TIN tubulointerstitium (vs. DKD). These findings were unanticipated given that TIN is often characterized by robust immune cell infiltration (45), whereas immune cell infiltration in DKD can be overlooked despite being a process with maladaptive inflammation (46).

In this study, T cell markers CD4 and CD8 were significantly higher in DKD kidney compartments. The role of T cells in the pathogenesis of DKD and associated proteinuria remains largely unclear. Bending et al. (20) found that the absolute numbers of activated T cells were significantly higher among DKD patients with microalbuminuria than diabetic patients without proteinuria (p<0.05). Additionally, Moon et al. (19) showed higher expression of IFN-γ secreted by CD3+ T cells isolated from streptozotocin-induced (Type 1) DKD mice kidney tissue. Similarly, in a case-control study, T cell mediators, including IFN-γ and TNF-α, were significantly higher in DKD patients than in normal kidney subjects (47). Consequently, these mediators activate macrophages, resulting in kidney infiltration, podocyte damage, and proteinuria development. *In vitro* studies showed activation of APCs under hyperglycemia conditions, leading to activation of T cells (48). Notably, blocking this activating interaction with abatacept, a fusion protein that targets the APC CD80 implicated in T cell activation, decreased infiltrating kidney T cells and significantly prevented albuminuria in diabetic mice (49). These studies suggest that targeting the direct cytotoxicity of T cells or the indirect activation of immune cells may hold promise in DKD prevention.

Our data showed a significant infiltration of both pro-inflammatory M1 (CD68+) and pro-repair M2 (CD163+) macrophage phenotypes in DKD interstitium and tubules compared to normal. This aligns with the growing evidence linking macrophage infiltration to DKD pathogenesis and progression. A recent study in DKD mice with overexpressed MCP-1 had significant macrophage infiltration compared to db/db control mice (50). Similarly, Fu et al. (51), using scRNA-seq analysis, reported substantial macrophage infiltration in Type I DKD mice glomeruli vs. control (1.5% vs. 15%) in DKD. Infiltrating macrophages express cytokines implemented in kidney injury and fibrosis (18). In our studies, CD68 macrophage marker was increased across all 3 DKD compartments (vs. normal), including the glomeruli, which had fewer overall differences in cell prevalence. Indeed, both glomerular and interstitial macrophage infiltration in Type 2 diabetic (db/db) mice significantly correlated with blood glucose levels, albuminuria, tubular atrophy, and interstitial fibrosis (17). Recently, CD68+ macrophage intersitium density predicted response to immunosuppression therapy in a glomerular disease (52) and may be relevant for future DKD therapy. To date, anti-macrophage therapy has shown promise but none are approved by the U.S. Food and Drug Administration for DKD (53). nuclear factor kappa-light-chain-enhancer of activated b cells (NF-κB), JAK/STAT, and NLRP3 inflammasome pathways fuel the pro-inflammatory environment in DKD. Successful development of safe therapies that effectively antagonize macrophage infiltration or reduce macrophage chemoattractants may limit the progression of DKD (54, 55).

Interestingly, in our study, the HSC and endothelial marker CD34 was lower in tubular and interstitial compartments of DKD vs. normal. CD34 is expressed in multiple stem/progenitor cells including HSC and endothelial progenitor cells (56). Tian et al. (57) also reported decreased expression of CD34+ cells via immunohistochemical staining on tubulointerstitial samples from patients with DKD. In addition, Makino et al. (58) found decreased levels of circulating CD34+ cells that were negatively associated with albuminuria. CD34+ cells may be decreased in DKD due to the toxic effects of uremia on bone marrow productivity (59). Consequently, decreased levels of CD34+ cells may reduce the neovascular regenerative integrity, disrupting the glomerular basement membrane and further contributing to DKD pathogenesis. CD34 is also used as an endothelial cell marker and to identify endothelial progenitor cells. In the tubulointerstitial compartment, CD34 will predominantly label the peritubular capillary endothelial cells. There is a correlation between peritubular capillary density and interstitial fibrosis, with several possible mechanistic explanations (60). In contrast to the tubulointerstitial compartments, we did not find differences in CD34+ levels in the glomeruli. However, Hou et al. and Acevedo et al. (61, 62) found CD34+ cells to be highly expressed in DKD rat glomeruli, which could indicate increased glomerular endothelium proliferation.

The study had limitations. Spatial transcriptomics studies in clinical research typically involve limited sample sizes due to the stringent need for high-quality biopsy material and the considerable cost and complexity of the technology. To improve output and test our hypothesis, we utilized 12 ROIs from each biopsy. Despite the small number of patient biopsies studied, data were sufficient to garner substantial evidence of differences between normal, DKD, and TIN biopsy groups. Furthermore, disease severity represents an important variable that may influence immune landscape heterogeneity across kidney diseases. The cohort size did not permit formal stratification or comparative analyses based on severity grading with adequate statistical power. However, all cases showed moderate interstitial fibrosis and tubular atrophy with associated interstitial inflammation. Future studies incorporating larger cohorts will be necessary to define how immune spatial organization evolves across distinct stages of DKD as compared to TIN and healthy controls. Additionally, DSP technology may have challenges with single cell resolution (30, 31). Nonetheless, these investigations uniquely capture large and complex data. In this process, we attained proof-of-concept and demonstrated feasibility which can inform larger studies. Thus, despite the potential limitations, this study provides an important contribution to the ongoing efforts to gain insight into the role of inflammation in DKD pathogenesis.

In conclusion, we found that T cells and macrophage infiltration are robust in DKD and has significant overlap with TIN. This provides further evidence for the need to develop novel therapeutics that target inflammation in DKD. Furthermore, NanoString GeoMx DSP technology may fulfila role in enhancing the understanding of DKD pathogenesis as well as measuring response to therapy. Future directions may include applying NanoString GeoMx DSP in larger biopsy studies of DKD to further elucidate the inflammatory landscape and identifying therapeutic targets for DKD.

## Data Availability

The original contributions presented in the study are included in the article/supplementary material. Further inquiries can be directed to the corresponding author.

## Supplementary material

Supplementary Figure 1 Regions of Interest (ROI) by kidney compartment and disease groups for Nanostring GeoMx digital spatial profiling in human kidney tissue. Representative images are shown. ROIs are obtained from each biopsy slide to capture glomerular and tubulointerstitial regions (left). Segmentation of tubules and interstitial compartments in disease groups for normal, DKD, and TIN kidney tissue (right). Segmentation of tubules (green): pan-cytokeratin positive and interstitium (pink): pan-cytokeratin negative, SYTO13 positive. DKD, diabetic kidney disease and TIN, tubulointerstitial nephritis; SYTO13: nucleic acid stain.

Supplementary Figure 2 Immune cell marker distributions in the glomerular compartments of Normal, DKD, and TIN kidney tissue. Forest plots display differential expression of cell marker proteins. Data are displayed as Log2 Fold Change (Log2FC) and 95% confidence intervals (CIs). Comparison analyses are shown for the glomerular compartments (DKD vs. NL [left] and DKD vs. TIN [right]). APC, antigen-presenting cell; DKD, diabetic kidney disease; Gran, granulocyte; HSC, hematopoietic stem cell; MΦ, macrophage; NK cell, natural killer Cell; NL, normal kidney tissue; and TIN, tubulointerstitial nephritis.

Supplementary Figure 3 T regulatory cell marker distributions in the glomerular, tubular and interstitial compartments of Normal, DKD, and TIN kidney tissue. Forest plots display differential expression of cell marker proteins. Data are displayed as Log2 Fold Change (Log2FC) and 95% confidence intervals (CIs). Comparison analyses are shown for DKD vs. NL [left] and DKD vs. TIN [right]. APC, antigen-presenting cell; DKD, diabetic kidney disease; Gran, granulocyte; HSC, hematopoietic stem cell; MΦ, macrophage; NK cell, natural killer cell; NL, normal kidney tissue; and TIN, tubulointerstitial nephritis.

## References

[1] Koye DN Magliano DJ Nelson RG Pavkov ME . The global epidemiology of diabetes and kidney disease. Adv Chronic Kidney Dis. (2018) 25:121–32. doi: 10.1053/j.ackd.2017.10.011, PMID: 29580576 PMC11000253

[2] Sun H Saeedi P Karuranga S Pinkepank M Ogurtsova K Duncan BB . IDF Diabetes Atlas: Global, regional and country-level diabetes prevalence estimates for 2021 and projections for 2045. Diabetes Res Clin Pract. (2022) 183:109119. doi: 10.1016/j.diabres.2021.109119, PMID: 34879977 PMC11057359

[3] Alicic RZ Rooney MT Tuttle KR . Diabetic kidney disease: challenges, progress, and possibilities. Clin J Am Soc Nephrol. (2017) 12:2032–45. doi: 10.2215/CJN.11491116, PMID: 28522654 PMC5718284

[4] de Boer IH . A new chapter for diabetic kidney disease. N Engl J Med. (2017) 377:885–7. doi: 10.1056/NEJMe1708949, PMID: 28854097

[5] Heerspink HJL Parving HH Andress DL Bakris G Correa-Rotter R Hou FF . Atrasentan and renal events in patients with type 2 diabetes and chronic kidney disease (SONAR): a double-blind, randomised, placebo-controlled trial. Lancet. (2019) 393:1937–47. doi: 10.1016/S0140-6736(19)30772-X, PMID: 30995972

[6] Alicic RZ Johnson EJ Tuttle KR . Inflammatory mechanisms as new biomarkers and therapeutic targets for diabetic kidney disease. Adv Chronic Kidney Dis. (2018) 25:181–91. doi: 10.1053/j.ackd.2017.12.002, PMID: 29580582

[7] Tesch GH . Macrophages and diabetic nephropathy. Semin Nephrol. (2010) 30:290–301. doi: 10.1016/j.semnephrol.2010.03.007, PMID: 20620673

[8] Patel HA Wang J Zinn CJ Learmonth M Lerman LO Wolfram J . Fortifying the diabetic kidney disease treatment armamentarium: multitarget senotherapeutic and regenerative strategies. J Am Soc Nephrol. (2025) 36:1655–8. doi: 10.1681/ASN.0000000754, PMID: 40333016 PMC12342067

[9] Agarwal R Green JB Heerspink HJL Mann JFE McGill JB Mottl AK . Finerenone with empagliflozin in chronic kidney disease and type 2 diabetes. N Engl J Med. (2025) 393:533–43. doi: 10.1056/NEJMoa2410659, PMID: 40470996

[10] Zheng Z Zheng F . Immune cells and inflammation in diabetic nephropathy. J Diabetes Res. (2016) 2016:1841690. doi: 10.1155/2016/1841690, PMID: 26824038 PMC4707326

[11] Bhatt K Lanting LL Jia Y Yadav S Reddy MA Magilnick N . Anti-inflammatory role of microRNA-146a in the pathogenesis of diabetic nephropathy. J Am Soc Nephrol. (2016) 27:2277–88., PMID: 26647423 10.1681/ASN.2015010111 PMC4978034

[12] Tang SCW Yiu WH . Innate immunity in diabetic kidney disease. Nat Rev Nephrol. (2020). doi: 10.1038/s41581-019-0234-4, PMID: 31942046

[13] Chow FY Nikolic-Paterson DJ Ozols RC Atkins BJ Rollin GH Tesch GH . Monocyte chemoattractant protein-1 promotes the development of diabetic renal injury in streptozotocin-treated mice. Kidney Int. (2006) 69:73–80. doi: 10.1038/sj.ki.5000014, PMID: 16374426

[14] Okada S Shikata K Matsuda M Ogawa D Usui H Kido Y . Intercellular adhesion molecule-1-deficient mice are resistant against renal injury after induction of diabetes. Diabetes. (2003) 52:2586–93. doi: 10.2337/diabetes.52.10.2586, PMID: 14514644

[15] Tsai YC Kuo PL Kuo MC Hung WW Wu LY Chang WA . The interaction of miR-378i-skp2 regulates cell senescence in diabetic nephropathy. J Clin Med. (2018) 7. doi: 10.3390/jcm7120468, PMID: 30469549 PMC6306775

[16] Fu J Sun Z Wang X Zhang T Yuan W Salem F . The single-cell landscape of kidney immune cells reveals transcriptional heterogeneity in early diabetic kidney disease. Kidney Int. (2022) 102:1291–304. doi: 10.1016/j.kint.2022.08.026, PMID: 36108806 PMC9691617

[17] Chow F Ozols E Nikolic-Paterson DJ Atkins RC Tesch GH . Macrophages in mouse type 2 diabetic nephropathy: correlation with diabetic state and progressive renal injury. Kidney Int. (2004) 65:116–28. doi: 10.1111/j.1523-1755.2004.00367.x, PMID: 14675042

[18] Ninichuk V Khandoga AG Segerer S Loetscher P Schlapbach A Revesz L . The role of interstitial macrophages in nephropathy of type 2 diabetic db/db mice. Am J Pathol. (2007) 170:1267–76. doi: 10.2353/ajpath.2007.060937, PMID: 17392166 PMC1829460

[19] Moon JY Jeong KH Lee TW Ihm CG Lim SJ Lee SH . Aberrant recruitment and activation of T cells in diabetic nephropathy. Am J Nephrol. (2012) 35:164–74. doi: 10.1159/000334928, PMID: 22286547

[20] Bending JJ Lobo-Yeo A Vergani D Viberti GC . Proteinuria and activated T-lymphocytes in diabetic nephropathy. Diabetes. (1988) 37:507–11. doi: 10.2337/diab.37.5.507, PMID: 3258834

[21] Tesch GH . Diabetic nephropathy – is this an immune disorder? Clin Sci. (2017) 131:2183–99. 10.1042/CS20160636 28760771

[22] Yan J Li X Liu N He JA Zhong Y . Relationship between macrophages and tissue microenvironments in diabetic kidneys. Biomedicines. (2023) 11:1889. doi: 10.3390/biomedicines11071889, PMID: 37509528 PMC10377233

[23] Liu Y Lv Y Zhang T Huang T Lang Y Sheng Q . T cells and their products in diabetic kidney disease. Front Immunol. (2023) 14. doi: 10.3389/fimmu.2023.1084448, PMID: 36776877 PMC9909022

[24] Chow FY Nikolic-Paterson DJ Atkins RC Tesch GH . Macrophages in streptozotocin-induced diabetic nephropathy: potential role in renal fibrosis. Nephrol Dialysis Transplant. (2004) 19:2987–96. doi: 10.1093/ndt/gfh441, PMID: 15574996

[25] Nguyen D Ping F Mu W Hill P Atkins RC Chadban SJ . Macrophage accumulation in human progressive diabetic nephropathy. Nephrol (Carlton). (2006) 11:226–31. doi: 10.1111/j.1440-1797.2006.00576.x, PMID: 16756636

[26] Matsui H Suzuki M Tsukuda R Iida K Miyasaka M Ikeda H . Expression of ICAM-1 on glomeruli is associated with progression of diabetic nephropathy in a genetically obese diabetic rat, Wistar fatty. Diabetes Res Clin Pract. (1996) 32:1–9. doi: 10.1016/0168-8227(96)01209-0, PMID: 8803476

[27] Wu H Gonzalez Villalobos R Yao X Reilly D Chen T Rankin M . Mapping the single-cell transcriptomic response of murine diabetic kidney disease to therapies. Cell Metab. (2022) 34:1064–1078.e6. doi: 10.1016/j.cmet.2022.05.010, PMID: 35709763 PMC9262852

[28] Lewis SM Asselin-Labat ML Nguyen Q Berthelet J Tan X Wimmer VC . Spatial omics and multiplexed imaging to explore cancer biology. Nat Methods. (2021) 18:997–1012. doi: 10.1038/s41592-021-01203-6, PMID: 34341583

[29] Bergholtz H Carter JM Cesano A Cheang MCU Church SE Divakar P . Best practices for spatial profiling for breast cancer research with the geoMx(^®^) digital spatial profiler. Cancers (Basel). (2021) 13. doi: 10.3390/cancers13174456, PMID: 34503266 PMC8431590

[30] Schoenfeld DA Merkin RD Moutafi M Martinez S Adeniran A Kumar D . Location matters: LAG3 levels are lower in renal cell carcinoma metastatic sites compared to primary tumors, and expression at metastatic sites only may have prognostic importance. Front Oncol. (2022) 12:990367. doi: 10.3389/fonc.2022.990367, PMID: 36313654 PMC9608089

[31] Hernandez S Lazcano R Serrano A Powell S Kostousov L Mehta J . Challenges and opportunities for immunoprofiling using a spatial high-plex technology: the nanoString geoMx(^®^) digital spatial profiler. Front Oncol. (2022) 12:890410. doi: 10.3389/fonc.2022.890410, PMID: 35847846 PMC9277770

[32] Ye L Liu Y Zhu X Duan T Wang C Fu X . Digital spatial profiling of individual glomeruli from patients with anti-neutrophil cytoplasmic autoantibody-associated glomerulonephritis. Front Immunol. (2022) 13:831253. doi: 10.3389/fimmu.2022.831253, PMID: 35309370 PMC8924137

[33] Oszwald A Mejía-Pedroza RA Schachner H Aigner C Rees A Kain R . Digital spatial profiling of glomerular gene expression in pauci-immune focal necrotizing glomerulonephritis. Kidney360. (2023) 4:83–91. doi: 10.34067/KID.000461202, PMID: 36700908 PMC10101620

[34] Carter JM Polley MC Leon-Ferre RA Sinnwell J Thompson KJ Wang X . Characteristics and spatially defined immune (micro)landscapes of early-stage PD-L1-positive triple-negative breast cancer. Clin Cancer Res. (2021) 27:5628–37. doi: 10.1158/1078-0432.CCR-21-0343, PMID: 34108182 PMC8808363

[35] Van Acker HH Capsomidis A Smits EL Van Tendeloo VF . CD56 in the immune system: more than a marker for cytotoxicity? Front Immunol. (2017) 8:892., PMID: 28791027 10.3389/fimmu.2017.00892 PMC5522883

[36] Dai DF Sasaki K Lin MY Smith KD Nicosia RF Alpers CE . Interstitial eosinophilic aggregates in diabetic nephropathy: allergy or not? Nephrol Dial Transplant. (2015) 30:1370–6., PMID: 25813275 10.1093/ndt/gfv067

[37] Houghton DC Troxell ML . An abundance of IgG4+ plasma cells is not specific for IgG4-related tubulointerstitial nephritis. Modern Pathol. (2011) 24:1480–7. doi: 10.1038/modpathol.2011.101, PMID: 21701536

[38] Markovic-Lipkovski J Müller CA Risler T Bohle A Müller GA . Association of glomerular and interstitial mononuclear leukocytes with different forms of glomerulonephritis. Nephrol Dial Transplant. (1990) 5:10–7. doi: 10.1093/ndt/5.1.10, PMID: 2109281

[39] Ditscherlein G . Renal histopathology in hypertensive diabetic patients. Hypertension. (1985) 7:II29. doi: 10.1161/01.HYP.7.6_Pt_2.II29, PMID: 4077238

[40] Lewis A Steadman R Manley P Craig K de la Motte C Hascall V . Diabetic nephropathy, inflammation, hyaluronan and interstitial fibrosis. Histol Histopathol. (2008) 23:731–9., PMID: 18366011 10.14670/HH-23.731

[41] Hooke DH Gee DC Atkins RC . Leukocyte analysis using monoclonal antibodies in human glomerulonephritis. Kidney Int. (1987) 31:964–72. doi: 10.1038/ki.1987.93, PMID: 3495689

[42] Lim AKH Tesch GH . Inflammation in diabetic nephropathy. Mediators Inflammation. (2012) 2012:146154. doi: 10.1155/2012/146154, PMID: 22969168 PMC3432398

[43] Furuta T Saito T Ootaka T Soma J Obara K Abe K . The role of macrophages in diabetic glomerulosclerosis. Am J Kidney Dis. (1993) 21:480–5. doi: 10.1016/S0272-6386(12)80393-3, PMID: 8488815

[44] Iványi B Hamilton-Dutoit SJ Hansen HE Olsen S . Acute tubulointerstitial nephritis: phenotype of infiltrating cells and prognostic impact of tubulitis. Virchows Arch. (1996) 428:5–12. doi: 10.1007/BF00192921, PMID: 8646369

[45] Tanaka T Nangaku M . Pathogenesis of tubular interstitial nephritis. In: S.Karger AG , editor. Experimental models for renal diseases: pathogenesis and diagnosis (2011). doi: 10.1159/000314577, PMID: 21252528

[46] Turkmen K . Inflammation, oxidative stress, apoptosis, and autophagy in diabetes mellitus and diabetic kidney disease: the Four Horsemen of the Apocalypse. Int Urol Nephrol. (2017) 49:837–44. doi: 10.1007/s11255-016-1488-4, PMID: 28035619

[47] Zhang C Xiao C Wang P Xu W Zhang A Li Q . The alteration of Th1/Th2/Th17/Treg paradigm in patients with type 2 diabetes mellitus: Relationship with diabetic nephropathy. Hum Immunol. (2014) 75:289–96. doi: 10.1016/j.humimm.2014.02.007, PMID: 24530745

[48] Iwai N Steib C Marzo A Lerret NM . The role of hyperglycemia in CD4 T cell survival and differentiation. Am Soc Clin Lab Sci. (2018) ascls.118.000331. doi: 10.29074/ascls.2018000331

[49] Herrera M Söderberg M Sabirsh A Valastro B Mölne J Santamaria B . Inhibition of T-cell activation by the CTLA4-Fc Abatacept is sufficient to ameliorate proteinuric kidney disease. Am J Physiology-Renal Physiol. (2017) 312:F748–59. doi: 10.1152/ajprenal.00179.2016, PMID: 27440778

[50] Zhang YY Tang PM Tang PC Xiao J Huang XR Yu C . LRNA9884, a Novel Smad3-Dependent Long Noncoding RNA, Promotes Diabetic Kidney Injury in db/db Mice via Enhancing MCP-1-Dependent Renal Inflammation. Diabetes. (2019) 68:1485–98. doi: 10.2337/db18-1075, PMID: 31048367

[51] Fu J Akat KM Sun Z Zhang W Schlondorff D Liu Z . Single-cell RNA profiling of glomerular cells shows dynamic changes in experimental diabetic kidney disease. J Am Soc Nephrol. (2019) 30:533–45. doi: 10.1681/ASN.2018090896, PMID: 30846559 PMC6442341

[52] Wang J Lou W Zhu M Tu Y Chen D Qiu D . Prediction of treatment response in lupus nephritis using density of tubulointerstitial macrophage infiltration. Front Immunol. (2024) 15:1321507. doi: 10.3389/fimmu.2024.1321507, PMID: 38415246 PMC10896899

[53] Menne J Eulberg D Beyer D Baumann M Saudek F Valkusz Z . C-C motif-ligand 2 inhibition with emapticap pegol (NOX-E36) in type 2 diabetic patients with albuminuria. Nephrol Dial Transplant. (2017) 32:307–15. doi: 10.1093/ndt/gfv459, PMID: 28186566 PMC5410979

[54] Zhao H Guo J . Macrophages in focus: key drivers and therapeutic opportunities in diabetic kidney disease. Int J Biol Sci. (2025) 21:4647–62. doi: 10.7150/ijbs.112737, PMID: 40765824 PMC12320494

[55] Ansari Z Chaurasia A Neha Sharma N Bachheti RK Gupta PC . Exploring inflammatory and fibrotic mechanisms driving diabetic nephropathy progression. Cytokine Growth Factor Rev. (2025) 84:120–34. doi: 10.1016/j.cytogfr.2025.05.007, PMID: 40467395

[56] Hassanpour M Salybekov AA Kobayashi S Asahara T . CD34 positive cells as endothelial progenitor cells in biology and medicine. Front Cell Dev Biol. (2023) 11:1128134. doi: 10.3389/fcell.2023.1128134, PMID: 37138792 PMC10150654

[57] Tian D Li J Zou L Lin M Shi X Hu Y . Adenosine A1 receptor deficiency aggravates extracellular matrix accumulation in diabetic nephropathy through disturbance of peritubular microenvironment. J Diabetes Res. (2021) 2021:5584871. doi: 10.1155/2021/5584871, PMID: 34671682 PMC8523293

[58] Makino H Okada S Nagumo A Sugisawa T Miyamoto Y Kishimoto I . Decreased circulating CD34+ cells are associated with progression of diabetic nephropathy. Diabetes Med. (2009) 26:171–3. doi: 10.1111/j.1464-5491.2008.02638.x, PMID: 19236621

[59] Krenning G Dankers PY Drouven JW Waanders F Franssen CF van Luyn MJ . Endothelial progenitor cell dysfunction in patients with progressive chronic kidney disease. Am J Physiol Renal Physiol. (2009) 296:F1314–22. doi: 10.1152/ajprenal.90755.2008, PMID: 19339628 PMC2692451

[60] Venkatachalam MA Weinberg JM Kriz W Bidani AK . Failed tubule recovery, AKI-CKD transition, and kidney disease progression. J Am Soc Nephrol. (2015) 26:1765–76. doi: 10.1681/ASN.2015010006, PMID: 25810494 PMC4520181

[61] Hou N Huang N Han F Zhao J Liu X Sun X . Protective effects of adiponectin on uncoupling of glomerular VEGF-NO axis in earlflammation in diabetic nephropaty streptozotocin-induced type 2 diabetic rats. Int Urol Nephrol. (2014) 46:2045–51. doi: 10.1007/s11255-014-0807-x, PMID: 25118612

[62] Acevedo LM Londono I Oubaha M Ghitescu L Bendayan M . Glomerular CD34 expression in short- and long-term diabetes. J Histochem Cytochem. (2008) 56:605–14. doi: 10.1369/jhc.7A7354.2008, PMID: 18319274 PMC2386768

