# Supplementary material for: Capturing the inflammatory landscape within kidney compartments of human diabetic kidney disease: a digital spatial profiling study
Source: Front Endocrinol (Lausanne). 2026 Feb 2;17:1744430. doi: 10.3389/fendo.2026.1744430 (PMC12907159; doi:10.3389/fendo.2026.1744430)

Supplemental Figure 1.

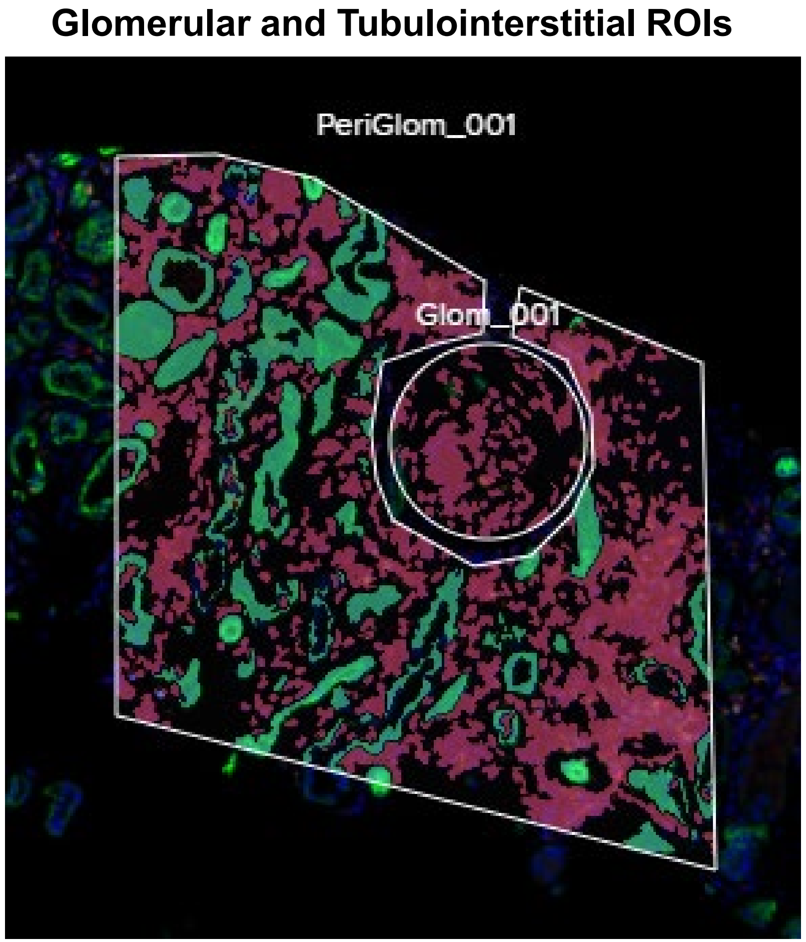

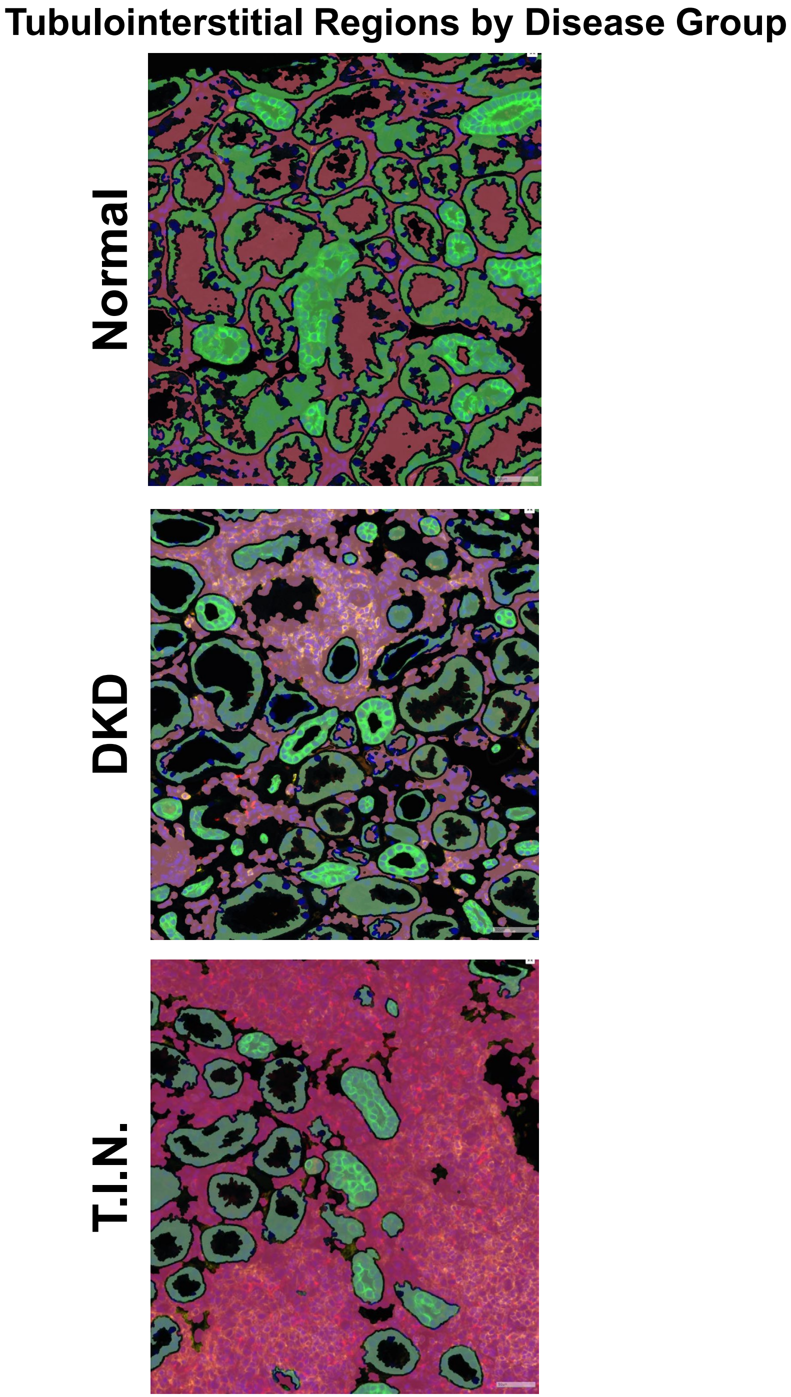

Supplemental Figure 2.

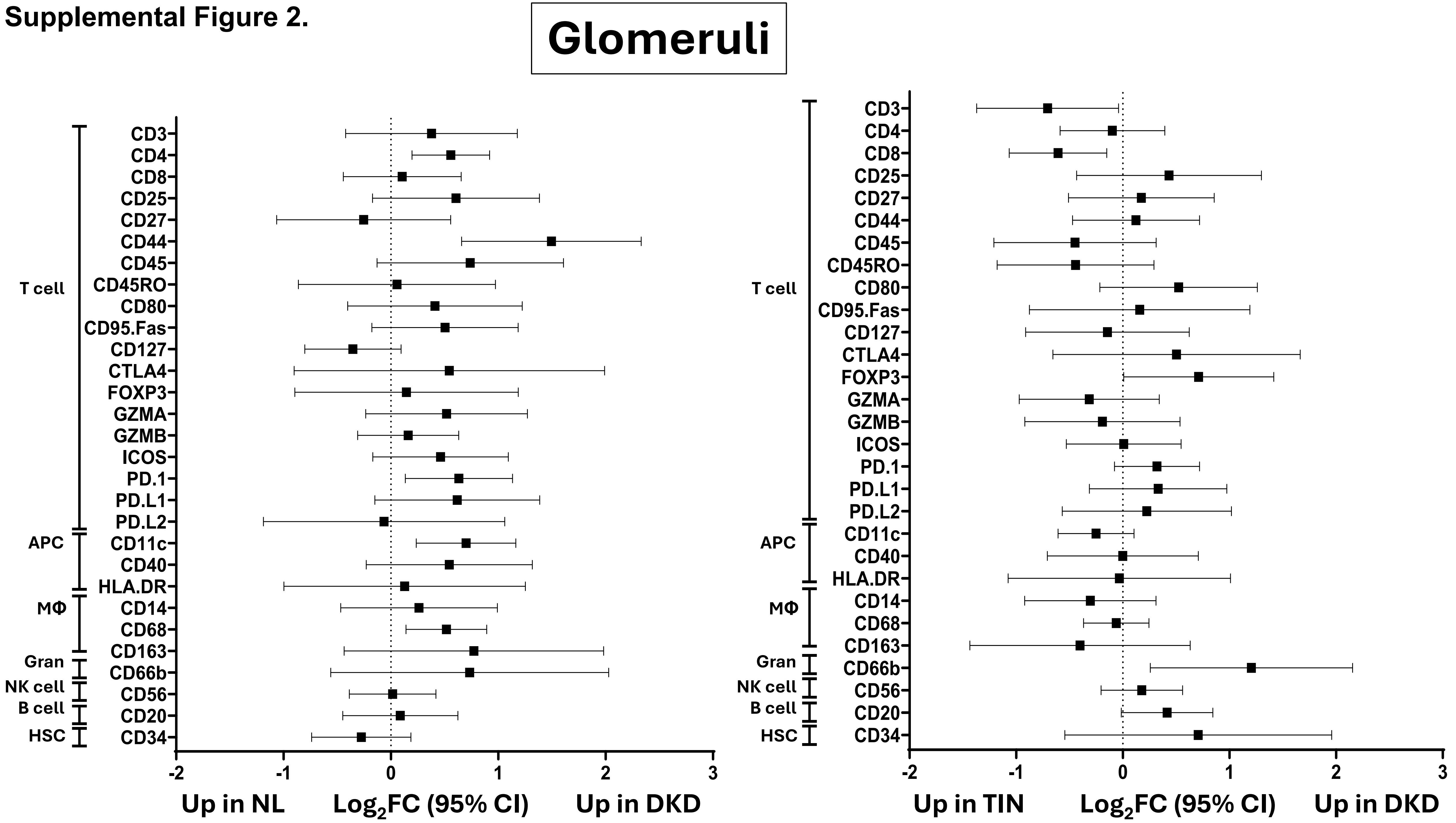

Supplemental Figure 3.

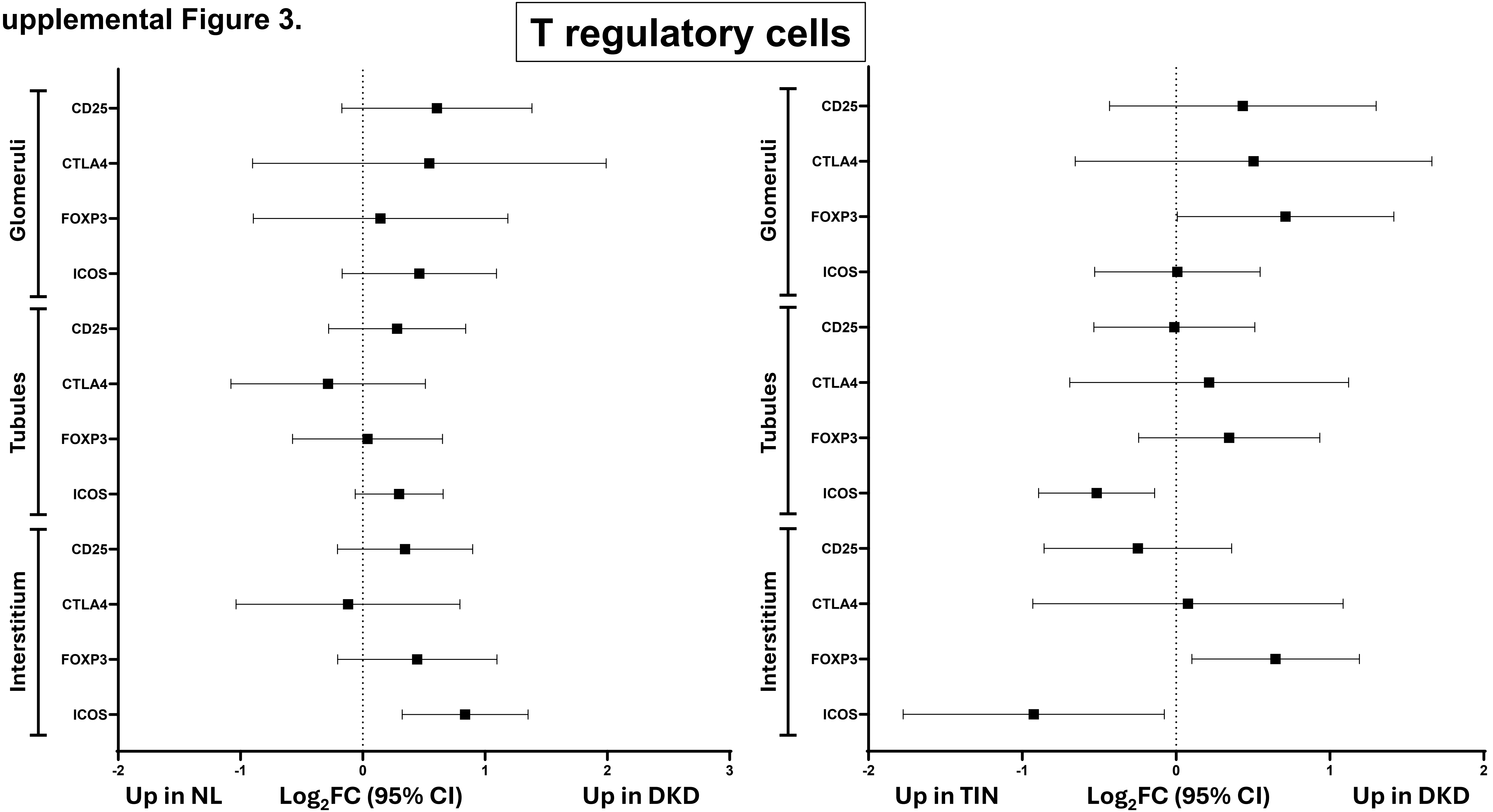

Supplement: Supplementary Figure 1 — Regions of Interest (ROI) by kidney compartment and disease groups for Nanostring GeoMx digital spatial profiling in human kidney tissue. Representative images are shown. ROIs are obtained from each biopsy slide to capture glomerular and tubulointerstitial regions (left). Segmentation of tubules and interstitial compartments in disease groups for normal, DKD, and TIN kidney tissue (right). Segmentation of tubules (green): pan-cytokeratin positive and interstitium (pink): pan-cytokeratin negative, SYTO13 positive. DKD, diabetic kidney disease and TIN, tubulointerstitial nephritis; SYTO13: nucleic acid stain. [file DataSheet1.pdf]
